# Incidence and risk factors associated with injuries during static line parachute training in Royal Thai Army

**DOI:** 10.1186/s40779-020-00252-w

**Published:** 2020-06-07

**Authors:** Watcharaphat Maneechaeye, Kathawoot Deepreecha, Wiroj Jiamjarasrangsi

**Affiliations:** 1 Armed Forces Research Institute of Medical Sciences, Royal Thai Army Medical Department, Bangkok, 10400 Thailand; 2 Health Promotion and Preventive Medicine Division, Royal Thai Army Medical Department, Bangkok, 10400 Thailand; 3 Department of Social and Preventive Medicine, Faculty of Medicine, Chulalongkorn University, Bangkok, 10330 Thailand

**Keywords:** Injuries, Parachute, Paratroopers

## Abstract

**Background:**

Incidence and risk factors of parachute injuries has been studied in developed countries, but not in trainees of the airborne forces in the Royal Thailand Army.

**Methods:**

A prospective cohort study was conducted among 992 military personnel who attended the basic airborne training program from February to July 2018. Information sheets were used to collect data about (a) personal demographics; (b) environmental conditions surrounding the parachute practice; and (c) parachute-related injuries. The incidence rate of injury was then calculated. Risk factors were examined using multilevel Poisson regression analysis and presented as incidence rate ratio (IRR) and 95% confidence interval (95% CI).

**Results:**

A total of 166 parachute-related injuries occurred in 4677 jumps. The incidence rate of injury was 35.50 per 1000 jumps (95%CI: 30.04–41.21). Factors significantly related to parachute injury included: jumping with equipment versus without equipment [adjusted IRR (95% CI): 1.28 (0.88–1.87)], higher wind speed [1.54 (1.27–1.87) per knot], airplane versus helicopter exit [1.75(0.68–4.55)], side versus rear exit [2.13 (1.43–3.23)], night versus day jumping [2.19 (0.81–5.90)], and presence of motion sickness [3.43 (1.93–6.92)].

**Conclusions:**

To prevent military static line parachute injuries, the following factors should be taken into consideration: type of aircraft, aircraft exit, time of the day, equipment, motion sickness and wind speed.

**Trial registration:**

The project was certified by the Research Ethics Committee, Faculty of Medicine, Chulalongkorn University (IRB No. 697/60).

## Background

Airborne operation by paratroopers is a critical part of modern warfare. It sends relatively large number of military personnel behind enemy lines. Parachuting requires extensive training. Injury and death during training are often, increase non-combat personnel loss and decrease troop morale. Factors associated with parachute injuries include aircraft (fixed wing vs. helicopter) and exit (side vs. rear) type, loading, timing (day vs. night), wind speed and temperature [1–5]. Incidents during parachute training in the Royal Thai Army have been reported in media, but no statistics are available. The Royal Thai Army conducts 5 to 6 parachute training classes each year, with 120 to 170 paratroopers per class. Each trainee received 5 static line parachute training sessions per class. Overall, 600 to 1000 paratroopers in the Royal Thai Army undergo a total of 3000–5000 jumps every year. Information regarding the incidence of, and factors associated with, static line parachute training is needed to design and optimize the training protocol, and to implement appropriate medical services by the Health Promotion and Preventive Medicine Division.

## Methods

### Data collection

This study was approved by the Research Ethics Committee, Faculty of Medicine, Chulalongkorn University (#697/60). Data were collected prospectively.

### Population and sample group

All trainees participating in the basic airborne training program at the Special Warfare School, Lopburi Province, Thailand, for the first time (without past parachuting experience) from February to July 2018 were screened.

### Inclusion criteria

All personnel at 18 to 60 years of age attending the basic airborne training program at the Special Warfare School from February to July 2018 were briefed and provided informed consent to participate in this study.

### Exclusion criteria

Personnel injured during ground training, those unable to jump, and those who were disqualified before the actual jump or those unwilling to participate in the research were excluded.

### Injury assessment

Data were collected using three forms. A General Information and Jump Record Form (Record Form A) was used to collect the following personal information prior to static line parachute training: age, weight, height, existing diseases, rank, jump history, and injuries from previous jumps. A Context of Jump Day Record Form (Record Form B) was used to collect environmental factors, including ground temperature, aircraft type and exit, load, time of the jump during the day, and surface of landing location. A Injuries After Jump Record Form (Record Form C) was used to record the injuries sustained during the parachute training; this form contained a checklist of location of the injury, severity (mild, moderate or severe injury, or death, as assessed upon triage) as classified by the standard textbook of emergency medicine [6] and treatment. Injury was defined as any bodily damage seen by the medic or medical personnel in the drop zone, from seating of the trainees in the aircraft to removal of the parachute harness after landing [1].

### Data analysis

All statistical analyses were performed using STATA software, version 14.0 (Stata, College Station, TX). Continuous variables, including age, weight, height, and body mass index (BMI, kg/m^2^), are presented as mean ± SD. Categorical variables are presented as frequency or percentage.

Injury was calculated based on jump number, and presented as events per 1000 jumps. A multi-level Poisson regression that compensated for non-independence of the data (multiple jumps by each trainee) was used to examine the factors associated with the injury. The risk is presented as incidence rate ratio (IRR) and 95% confidence interval. Bivariate analysis was used to determine the relation among independent variables. Two models were used. In Model 1, *P <* 0.25 was used to select factors [7]. The bivariate analysis result and backward elimination were then used to select the factors for the model at *P <* 0.10 as the criteria for sorting the factors. In Model 2, *P* was < 0.05 for factors that affected jumping from the related study and possible factors of practice (helicopter, jumping at night, rear exit, jumping with loading, motion sickness, and wind speed).

## Results

### Sample group characteristic

A total of 1026 trainees (four classes) attended the training program during a period from February to July 2018. Thirty-four were disqualified prior to the jump. The final analysis included 992 trainees (23.35 ± 3.76 years of age; Table 1). Majority of the trainees (76.41%) were non-commissioned officers. The average height was 171.23 ± 5.35 cm. The average weight was 65.18 ± 6.83 kg. Average BMI was 22.22 ± 1.94 kg/m^2^. Twenty-nine subjects (2.92%) had underlying diseases, including asthma, unspecified headache, dyspepsia and glucose-6-phosphate dehydrogenase (G6PD).

**Table 1:** Demographic data of the airborne trainees (*n* = 992)

Demographic data	Value
Age (year, *x* ± *s*)	23.35 ± 3.76
Rank [*n* (%)]	
Officer	35(4.84)
NCO	750(76.41)
Cadet	207(18.75)
Underlying diseases [*n* (%)]	
Without underlying disease	963(97.08)
With underlying disease	29(2.92)
Asthma	14(48.28)
Dyspepsia	7(24.14)
G6PD deficiency	5(17.24)
Others(Unspecific headache)	3(10.34)
Biometric measurement	
Height (cm, *x* ± *s*)	171.23 ± 5.35
Weight (kg, *x* ± *s*)	65.18 ± 6.83
Body mass index (kg/m^2^, *x* ± *s*)	22.22 ± 1.94

G6PD: Glucose-6-phosphate dehydrogenase; NCO: Noncommissioned officer

### Injuries

**Fig. 1:**
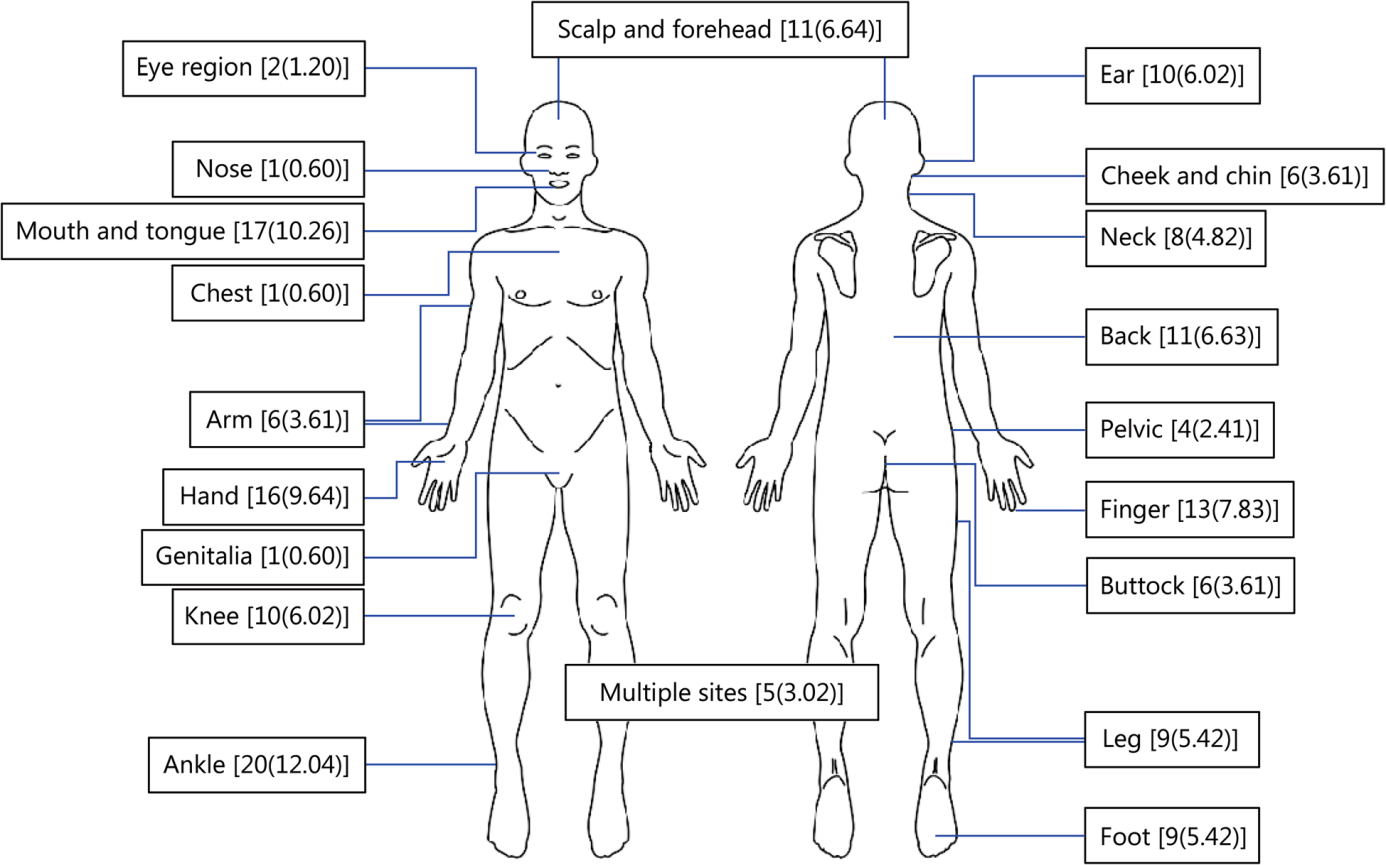
The airborne trainees injury site data [*n* (%)]

The rate of injury was 35.50 per 1000 jumps, with 95% CI at 30.04–41.21 (166 events in a total of 4677 jumps). The most common type of injury was abrasion and laceration, involving 92 trainees (55.42% of all events). Altogether 160 events had mild injuries and only needed only basic medical treatment by a medic (96.39% of all injuries) (Table 2). The top 4 injury sites are the ankle (12.04%; 20 events), mouth and tongue (10.25%; 17 events), hands (9.64%; 16 events) and fingers (7.83%; 13 events (Table 3 and Fig. 1).

**Table 2:** Injury data of the airborne trainees (*n* = 166 injuries)

Injury data	n (%)
Type of injury	
Contusion	29(17.47)
Sprain and strain	43(25.90)
Wound	92(55.42)
Fracture	2(1.21)
Severity	
Mild	160(96.39)
Moderate	6(3.61)
Treatment	
First aid	160(96.39)
Referral	6(3.61)

**Table 3:** Injury site data of the airborne trainees (*n* = 166 injuries)

Injury site	n(%)
Eye region	2(1.20)
Scalp and forehead	11(6.64)
Ear	10(6.02)
Nose	1(0.60)
Cheek and chin	6(3.61)
Mouth and tongue	17(10.26)
Neck	8(4.82)
Chest	1(0.60)
Back	11(6.64)
Arm	6(3.61)
Pelvis	4(2.41)
Hand	16(9.64)
Finger	13(7.83)
Genitalia	1(0.60)
Buttocks	6(3.61)
Knee	10(6.02)
Leg	9(5.42)
Ankle	20(12.04)
Foot	9(5.42)
Multiple sites	5(3.02)

The rate of injury differed significantly in the following factors: aircraft exit (side), loading and motion sickness (*P <* 0.01, Table 4).

**Table 4:** Incidence rate of military static line parachute injuries stratified by each factor

Factors	Jumps (*n*)	Injuries (*n*)	Incidence rate (/1000)		*P*-value
			Incidence	95%CI	
Type of aircraft					0.10
Fixed-wing airplane	3646	138	37.85	32.11–44.56	
Helicopter	1031	28	27.16	18.70–39.12	
Aircraft exit					< 0.01
Side exit	927	50	53.94	41.04–70.52	
Rear exit	3750	116	30.93	25.83–36.99	
Time of the day					0.70
Day	4074	143	35.10	29.86–41.22	
Night	603	23	38.14	25.28–56.85	
Jump with equipment					< 0.01
Without	3686	115	31.20	26.04–37.34	
With equipment	991	51	51.46	39.25–67.14	
Motion sickness					< 0.01
No	4554	152	33.38	28.53–39.01	
Yes	123	14	113.82	67.86–183.22	
Wind speed					0.167
< 5 knots	2019	63	31.20	24.41–39.77	
≥ 5 knots	2658	103	38.75	32.02–46.81	
Temperature					0.718
< 32 °C	2740	95	34.67	28.42–42.23	
≥ 32 °C	1937	71	36.65	29.11–46.03	
Age (year)					0.801
< 20	167	4	23.95	6.52–61.33	
20–24	3254	118	36.26	30.02–43.43	
24–29	929	31	33.37	22.67–47.36	
≥ 29	327	13	39.76	21.17–67.98	
Body mass index (kg/m^2^)					0.484
< 23.00	3162	108	34.16	28.01–41.23	
23.00–25	1149	41	35.68	25.61–48.41	
≥ 25.00	366	17	46.45	27.06–74.37	
Rank					0.075
Officer	163	7	42.94	17.27–88.48	
NCO	3480	111	31.90	26.24–38.41	
Cadet	1034	48	46.42	34.23–61.55	
Total	4677	166	35.50	30.04–41.21	

NCO: Noncommissioned officer

### Factors associated with injury

Bivariate analysis indicated association between injuries with the following factors: aircraft type (fixed-wing airplane versus helicopter exit type (side versus rear), presence of motion sickness and higher wind speed under a linear model. In comparison < 5 knots, wind speed at ≥5 knots had one increased Celsius degree under a linear model. In Model 1 of the multivariate analysis, the factors associated with injury included airplane versus helicopter jump [adjusted IRR (95% CI) = 3.70 (1.47–9.09)], side versus rear exit [1.79 (1.25–2.56)], day versus night jumping [2.81 (1.07–7.41)] and presence of motion sickness [3.55 (2.00–6.31)]. In Model 2, the factors associated with injury included jumping with versus without loading [1.28 (0.88–1.87)], higher wind speed [1.54 for every knot, (1.27–1.87)], airplane versus helicopter jump [1.75 (0.68–4.55)], side versus rear exit [2.13 (1.43–3.23)], night versus day jumping [2.19 (0.81–5.90)] and presence of motion sickness [3.43 (1.93–6.92)] (Table 5).

**Table 5:** Incidence rate ratio (IRR) of the correlations between parachute injuries and other factors

Factors	Crude IRR	95%CI	Adjusted IRR1^a^	95%CI	Adjusted IRR2^b^	95%CI
Type of aircraft						
Plane	2.17^****^	1.45–3.23	3.70^***^	1.47–9.09	1.75	0.68–4.55
Helicopter	1.00	Reference	1.00	Reference	1.00	Reference
Aircraft exit						
Side exit	2.22^****^	1.59–3.13	1.79^***^	1.25–2.56	2.13^****^	1.43–3.23
Rear exit	1.00	Reference	1.00	Reference	1.00	Reference
Time of day						
Night	0.69	0.44–1.07	2.81^*^	1.07–7.41	2.19	0.81–5.90
Day	1.00	Reference	1.00	Reference	1.00	Reference
Jump with equipment						
With equipment	1.19	0.85–1.65			1.28	0.88–1.87
Without	1.00	Reference			1.00	Reference
Motion sickness						
Yes	4.47^****^	2.52–7.91	3.55^****^	2.00–6.31	3.43^****^	1.93–6.12
No	1.00	Reference	1.00	Reference	1.00	Reference
Wind speed (knot) Each 1 knot increase in wind speed	1.58^****^	1.35–1.85			1.54^****^	1.27–1.87
≥ 5	1.58^***^	1.15–2.18				
< 5	1.00	Reference				
Temperature (°C) Each 1 °C increase in temperature	1.18^****^	1.07–1.31				
≥ 32	1.06	0.78–1.44				
< 32	1.00	Reference				
Rank						
NCO	0.75	0.33–1.67				
Cadet	1.04	0.45–2.40				
Officer	1.00	Reference				
Body mass index (kg/m^2^)						
≥ 25.00	1.34	0.78–2.30				
23.00–25.00	1.05	0.72–1.52				
< 23.00	1.00	Reference				
Age (years)						
≥ 29	1.53	0.48–4.86				
24–29	1.29	0.44–3.77				
20–24	1.36	0.49–3.80				
< 20	1.00	Reference				

*IRR* Incidence rate ratio

* *P*< 0.05; **P< 0.01; ***P< 0.005; **** *P<*0.001

^a^Factors included in the regression model are helicopter, rear exit, night jump and motion sickness

^b^Factors included in the regression model are helicopter, rear exit, night jump, motion sickness, jump with equipment and high wind speed (assuming that wind speed and injuries have a linear correlation)

## Discussion

This research revealed an incidence of 35.50 injuries/1000 jumps for parachuting, and no deaths occurred. Related studies have reported an incidence of 7.1 to 50.5 injuries/1000 jumps [1, 8–11] (Table 6) and a death rate of 0.25/1000 jumps [9, 12]. Obviously, the incidences in this research were higher than those overseas and of those during World War II, which totaled only 21 injuries/1000 jumps [13]. However, this study was focused on 1) static line parachuting and 2) programs for those without experience in basic airborne training.

**Table 6:** Comparison of incidence rate of military parachute injuries

Investigation	Study design	Injury case definition	Sample	Type of parachuting	Incidence rate (/1000)
Knapik et al. [2], 2011	Prospective study	Physical damage to body reported by medics in the drop zone	The 82nd Airborne Division of the XVIII Airborne Corps; trained paratroopers in training exercises	Static line	10.5
Hughs et al. [4], 2008	Retrospective study	Injuries recorded in unit medical records	4th Battalion Royal Australian Regiment; trained paratroopers in training exercises	Static line	50.5
Farrow [5], 1992	Prospective study	Physical damage to body requiring evacuation from drop zone, withdrawal from exercise, duty restriction, or hospitalization (excluded abrasions and lacerations)	Parachute Battalion Group, Australia; trained paratroopers in training exercises	Static line	7.1
Dhar [6], 2007	Retrospective study	Referred cases for parachute related injuries from local military unit	Not clearly stated	Not clearly stated	Minor injuries = 13.5 Major injuries = 9.0
Deaton and Roby [7], 2010	Prospective study	Any personnel reporting to the military surgeon for care related to airborne operations in the drop zone and placed on limited duty	US Marine Reconnaissance unit in Iraq; trained paratroopers in training exercises	Static line	8.23
Essex-Lopresti [9], 1946	Retrospective study	Any physical damage recorded in drop zone by medical officer	6th Airborne Division, United Kingdom; trained paratroopers in training exercises	Static line	21.0

The researcher hypothesized that the higher injury incidence in this research was due to the higher temperature in Thailand than in other countries. The studies of Knapik et al. [1–3] reported that a temperature higher than 26 °C affected injury. Our research was conducted between the summer and rainy season, when the average temperature was 31.36 °C, which was higher than that in other studies conducted in colder areas. Another potential cause was the number of paratroopers jumping from the aircraft. Knapik et al. [3] revealed that a large number of paratroopers jumping at one time (more than 23 personnel) increased the risk of injury. The jumps in this research involved 40 to 50 paratroopers on the aircraft. Thus, this might have constituted one of the causes of the high rate of injury. Furthermore, this research defined injury, referring to the study of Knapik et al. [1], meaning any injury to the body including pain and bruises. The definition covering pain and bruises might be another reason the incidence was higher than in other research studies. The Thai Army applied the same landing technique as the US Army, as published by Bricknell et al. [4].

Factors significantly associated with parachute injuries included aircraft exit (side exit) and jumping with loading. This was in line with the research of Knapik et al. [1–3], Bricknell et al. [4], and Hay et al. [5], including the epidemiological report of the US Army 2010 (14). Factors with no relation to the study included type of aircraft, time of day (night/day), wind speed and temperature. However, this study found that motion sickness was associated with parachute injuries, which was not found in other studies.

The researcher found that the association of injury incidence with aircraft type and time of day, which were unrelated to injury incidence in other studies, might have resulted from the flight operation. Personnel conducted airplane flights five times more frequently than helicopter flights. This was also related to time of day. Personnel jumped during the day nine times more frequently than during the night. Differences in the number of jump sunder each condition affected the statistical analysis results, leading to similar injury incidences. Because this research was conducted at the end of summer and beginning of the rainy season, the average temperature, as well as the wind speed, did not differ much each day. All jumps in this research were conducted at Tha Due Parachute Field, Lopburi Province, which had calm winds, so wind speed rarely differed each day. The insignificance of temperature and wind speed might be the reason why they have no relation to injury from parachuting.

This research applied multi-level Poisson regression and obtained 2 models. Model 1 contained the factors associated with the injury, including type of aircraft, aircraft exit, time of day and presence of motion sickness. Helicopter and rear exit minimized the injuries from jumping. Referring to information in the epidemiological report of the US Army 2010 [14], jumping from the rear exit allowed the paratrooper to have more space, so posture was more appropriate. Moreover, to jump from the rear exit, the paratrooper must run straight but in an oblique line to jump from the side exit. The side exit space was limited, so posture was inappropriate. In addition, the rear exit was wider and presented no barrier, whereas the side exit was narrower with external barriers, such as an airplane wing, propeller and jet engine, which might affect the jump. Considering the aircraft, because the speed of a helicopter is slower than that of an airplane and jumping from a large helicopter only allowed the use of the rear exit, jumping from a helicopter resulted in fewer injuries than jumping from an airplane. Jumping at night caused injury because of limited visibility. Motion sickness affected the decision making of the paratrooper when floating in the air, so it impacted the selection of a safe landing spot and appropriate posture leading to injury. Model 2 contained potential factors from related overseas studies, so more factors were added from Model 1 (i.e., jumping with loading and wind speed). The research of Knapik et al. [1–3], Bricknell et al. [4], and Hay et al. [5], including the epidemiological report of the US Army 2010 [14], identified that jumping with loading and wind speed affected the incidence of injuries because loading involved the back and front as well as the leg and hip, making it difficult to land with an appropriate posture.

In short, the researcher regarded that, according to the statistical principle, Model 1 was appropriate in theory. However, based on related studies and observation of real situations, Model 2 provided theoretical and practical appropriateness.

### Limitations

This research had three limitations. First, some reports were subjective. Therefore, it was difficult to figure out if the injuries truly occurred. Second, the time and context were limited. This research was conducted at the end of summer and beginning of rainy season, so the data collection period was quite short. Thus, the associated factors were not entirely accurate. Moreover, training budgets differed with respect to aircraft use and fuel for each model. Last, jumping times differed depending on whether the situation was safe or not.

### Suggestions

This research illustrates that wind speed is controllable, which means that wind speed can be measured and predicted by the trainer. When strong winds are expected on the training day, jumping should be reconsidered. In addition, motion sickness can be readily prevented by medications. However, type of aircraft, aircraft exit, jumping time and loading are controllable factors. On the other hand, these factors cannot be adjusted in a real battle situation.

The researcher suggested further research should have a longer data collection period to determine differences between more factors. Moreover, the diagnosis of injury from parachuting should be more precise and clearer. Furthermore, studying other armed forces or contexts should be considered to explore a greater variety of factors.

As the most frequently injured sites included the ankle, hand and head, equipment to minimize these injuries (such as an ankle brace [15, 16]) should be designed to better suit parachuting and prevent injuries.

#### Conclusions

To prevent injuries from military static line parachuting, type of aircraft, aircraft exit, jump timing, equipment, motion sickness and wind speed should be considered.

## Supplementary information

**Additional file 1.**

## Data Availability

The datasets used and/or analyzed during the current study are available from the corresponding author on reasonable request.

## Abbreviations

BMI: Body mass index
G6PD: Glucose-6-phosphate dehydrogenase
IRR: Incidence rate ratio
NCO: Noncommissioned officer
