# Supplementary material for: Incidence and risk factors associated with injuries during static line parachute training in Royal Thai Army
Source: Mil Med Res. 2020 Jun 7;7:27. doi: 10.1186/s40779-020-00252-w (PMC7278130; doi:10.1186/s40779-020-00252-w)
Supplement: Supplementary file 1 — Additional file 1. [file 40779_2020_252_MOESM1_ESM.zip › Data coding Manual-Confirmed the deleted part.docx]

| No. | Variable | Type | Description | Using |
| --- | --- | --- | --- | --- |
| 1 | ID | String | ID of sample | Data sorting |
| 2 | Round | Scale | Class | Data sorting |
| 3 | Date | Date | Date written on 3 digits such as 142 = 14 Feb 2018 | Data sorting |
| 4 | Jump | Scale | Round of jumping | Factor |
| 5 | Age | Scale | Age (yrs.) | Demographic data |
| 6 | Weight | Scale | Weight (kg.) | Demographic data   Factor |
| 7 | Height | Scale | Height (cm.) | Demographic data   Factor |
| 8 | UDisease | Nominal | Underlying disease 0 = no 1 = yes | Demographic data   Factor |
| 9 | Injury | Nominal | Parachute injury 0 = no 1 = yes | Incidence Regression dependent VA |
| 10 | Diagnosis | Nominal | Diagnosis 0 = none  1 = contusion 2 = sprain and atrain 3 = wound 4 = dislocation of bone or joint 5 = fracture 6 = faint 7 = death | Descriptive |
| 11 | Severity | Ordinal | srverity 1 = minimal 2 = moderate 3 = severe 4 = death 0 = none | Descriptive |
| 12 | Heal | Ordinal | treatment 1 = first aid 2 = refer 0 = none | Descriptive |
| 13 | PastInjury | Nominal | Past jump injuries or not 0 = no 1 = yes | Factor |
| 14 | Field | Nominal | Drop zone 1 = soil 2 = sand 3 = grassland 4 = water | Factor |
| 15 | Vehicle | Nominal | Type of aircraft 1 = plane 2 = helicopter | Factor |
| 16 | Exit | Nominal | Jump exit 1 = side 2 = rear | Factor |
| 17 | WindSpeed | Scale | ความเร็วลม (น็อต) เฉลี่ยต่อวัน | Factor |
| 18 | Temperature | Scale | อุณหภูมิ (เซลเซียส) เฉลี่ยต่อวัน | Factor |
| 19 | Timing | Nominal | Parachuting timing 1 = Day 2 = night | Factor |
| 20 | Equipment | Nominal | Jump with backpack 0 = without 1 = with | Factor |
| 21 | Dizziness | Nominal | airsickness 0 = no 1 = yes | Factor |
| 24 | BMI | Scale | Body mass index | Demographic data /  Factor |
| 25 | Rank | nominal | rank  1 = officer  2 = NCO  3 = cadet | Demographic data /  Descriptive /  Factor |
| 26 | BMI group | nominal | Group of BMI  1 = BMI < 20.80  2 = BMI 20.81 – 22.10  3 = BMI 22.11 – 23.39  4 = BMI >=23.40 | Demographic data /  Descriptive /  Factor |
| 27 | Age group | nominal | Group of age  1 = < 19 ปี  2 = 20 – 24 ปี  3 = 25 – 29 ปี  4 = >= 30 ปี | Demographic data /  Descriptive /  Factor |
| 28 | Windspeed group | nominal | Group of windspeed  1 = < 5 knot  2 = >= 5 knot | Demographic data /  Descriptive /  Factor |
| 29 | Temperature group | nominal | Group of temperature  1 = < 32 C  2 = >= 32 C | Demographic data /  Descriptive /  Factor |
