# Supplementary material for: Incidence and risk factors associated with injuries during static line parachute training in Royal Thai Army
Source: Mil Med Res. 2020 Jun 7;7:27. doi: 10.1186/s40779-020-00252-w (PMC7278130; doi:10.1186/s40779-020-00252-w)
Supplement: Supplementary file 1 — Additional file 1. [file 40779_2020_252_MOESM1_ESM.zip › RecordForms(ENG).doc]

**(A):** **General data and jumping history record form**

ID …………………………………………

1. weight...............kg. height...............cm.
2. underlying diseases ( ) no ( ) yes .............................................................................
3. age ..................... years

Jumping history

| Date | Jump No. | No injury | Injury | Severity | Rx | Outcome of Rx (can or cannot jump) |
| --- | --- | --- | --- | --- | --- | --- |
|  | 1 |  |  | O minor  O major  O death | O first aid O refer |  |
|  | 2 |  |  | O minor  O major  O death | O first aid O refer |  |
|  | 3 |  |  | O minor  O major  O death | O first aid O refer |  |
|  | 4 |  |  | O minor  O major  O death | O first aid O refer |  |
|  | 5 |  |  | O minor  O major  O death | O first aid O refer |  |

**(B): Associated factors record form**

Class...........................Date................ Jump No. ( 1	2	3	4	5 )

| Stick No. | Type of aircraft | Exit | Stagnant  or entanglement | Wind speed (knot) | temperature  (degree Celsius) | Day or night | Equipment  Jump  (yes/no) |
| --- | --- | --- | --- | --- | --- | --- | --- |
| 1 |  |  |  |  |  |  |  |
| 2 |  |  |  |  |  |  |  |
| 3 |  |  |  |  |  |  |  |
| 4 |  |  |  |  |  |  |  |
| 5 |  |  |  |  |  |  |  |
|  |  |  |  |  |  |  |  |
|  |  |  |  |  |  |  |  |
|  |  |  |  |  |  |  |  |
|  |  |  |  |  |  |  |  |
|  |  |  |  |  |  |  |  |
|  |  |  |  |  |  |  |  |
|  |  |  |  |  |  |  |  |
|  |  |  |  |  |  |  |  |
|  |  |  |  |  |  |  |  |
|  |  |  |  |  |  |  |  |

**(C): Injuries checklist and injuries data record form**

ID...........................Date................ Jump No. ( 1	2	3	4	5 )

**Checklist and physical examination**

1. Can paratrooper report at drop zone?

O Yes

O No (If cannot, go to physical examination part)

2. Ask for air sick symptom during jumping.

O Airsick

O No airsick

3. Ask for injured part.
   O Not injured	O Injured

physical examination	physical examination
O head O torso O back
O arm	O leg	O pelvis

**Injuries data record**

**O No injuries**
**O Injuries**

**Type of injuries**
 O contusion
 O sprain/strain
 O wound
 O dislocation of bone or joint
 O fracture
 O sign of intra-abdominal hemorrhage
 O faint
 O death **Severity***
 O green	O yellow	O red	O black

**Rx**
O first aid	O refer

Dr.................................................................

*** severity (mild injury, moderate injury, severe injury, and death based on triage) as defined by the standard textbook of emergency medicine *(Tintinalli EJ, Stapczynski SJ, Ma JM, Yealy MD, et al. Disaster Preparedness. In: Tintinalli EJ. Tintinalli's Emergency Medicine: A Comprehensive Study Guide. 8th ed. New York: McGraw-Hill; 2016. p.29-30.)***
